# A rare case report: SCARF syndrome

**DOI:** 10.1002/ccr3.61

**Published:** 2014-03-16

**Authors:** Masoume Rahimpour, Mohammad bager Sohrabi, Sulmaz Kalhor, Hossein ali Khosravi, Poone Zolfaghari, Elahe Yahyaei

**Affiliations:** Shahroud University of Medical ScienceShahroud, Iran

**Keywords:** Rare disease, SCARF syndrome, Shahroud

## Abstract

**Key Clinical Message:**

SCARF syndrome is a very rare syndrome that so far only two cases have been reported in the papers. In this article, a 3-month-old female who exhibited SCARF syndrome presented with multiple congenital abnormalities and problems at Imam Hossein hospital of Shahroud.

## Introduction

SCARF syndrome is the name of a disease that is largely inherited and is transmitted as a recessive gene and dominant gene [1]. This syndrome is very rare and the incidence is less than 1 in a million newborn. Symptoms of this syndrome are skeletal problems, loose skin (cutis laxa), ambiguous genitalia, mental retardation, abnormal form of face, and the patient seems as if they have suffered from premature aging [1,2]. When we drag the skin of these children, it shows resilience and hangs but gradually returns to its initial state [3]. Baby not only takes an old man or old woman face but also may suffer from shortness of breath, abdominal hernia or hernia of the abdominal wall skin (because of loose skin), heart disorders, joint disorders, and particularly multiple dislocations [4,5]. Elastic fibers constitute ∼2% of overall skin tissue series and gives the skin elasticity and in these patients, this amount is less than 2% and functionality is disrupted. The other fibers in the skin called collagen fibers are stranded and constitute 70% of the skin tissues. These fibers give the skin its strength [1,3]. Both elastic and collagen fibers in the cutis laxa have problem and in amount and function, they are considered abnormal [6,7].

## Introduction of Patient

Patient was a 3-month-old female infant, with cough, fever, and shortness of breath (weight of 4200 g, height of 55 cm, and head circumference of 37.5 cm) was admitted and had been hospitalized with a diagnosis of aspiration pneumonia. Because of abnormal form of face, skin wrinkles and ambiguous genital system she was suspected of diagnosis of genetic disorders and was under investigation. In the patient's history, the child was delivered vaginally with a gestational age of 38 weeks that after birth because of poor feeding and lethargy was treated in her birthplace hospital for 1 week. The patient's parents were cousins. Pregnancy care was completed without any medication. Also, there was no family history of similar disease and the infant was the first child of the family.

**Figure 1:**
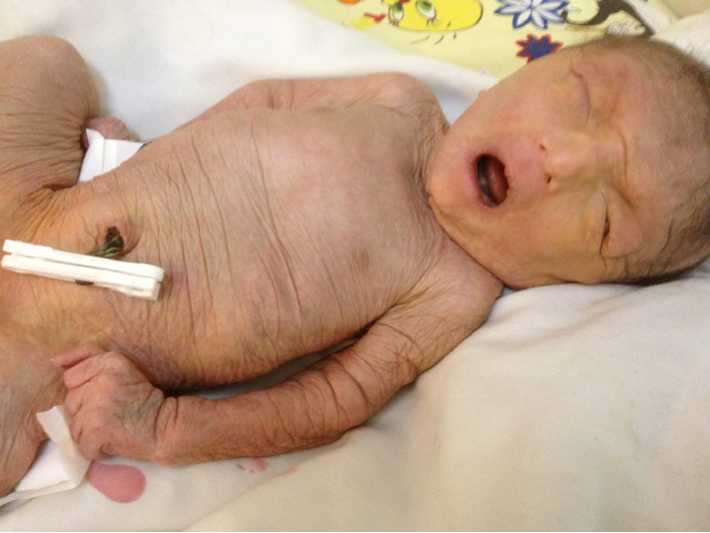
Fase abnormality.

**Figure 2:**
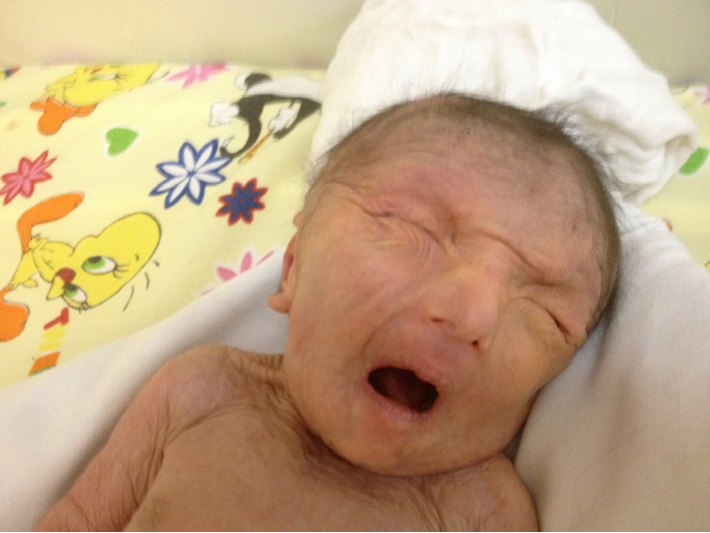
Fase abnormality and deformity.

**Figure 3:**
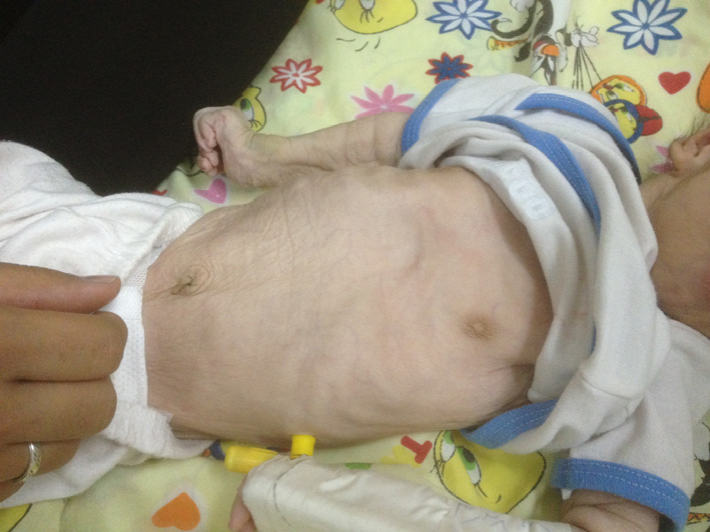
Abnormal abdominal shape.

**Figure 4:**
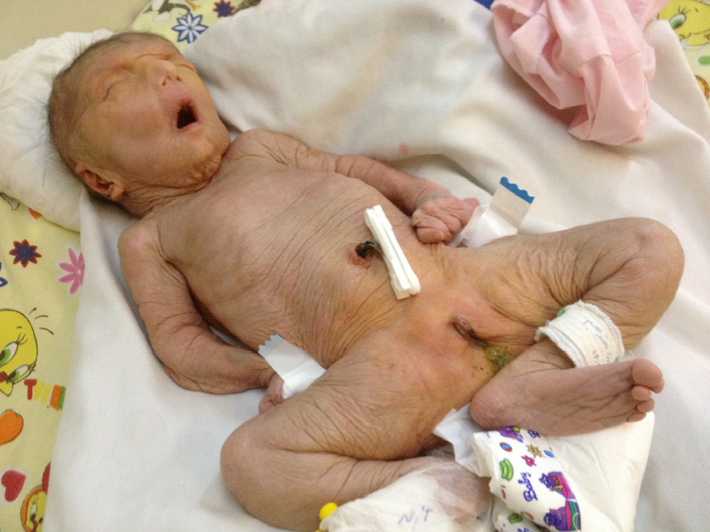
Ambiguous genitalia.

**Figure 5:**
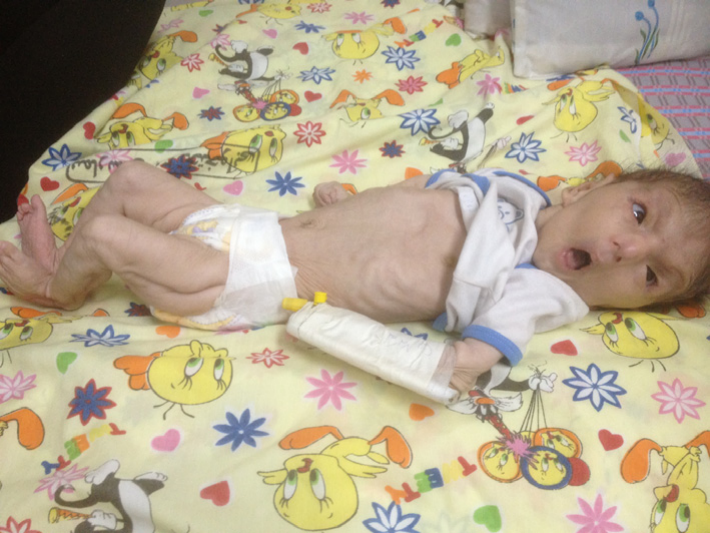
Extremity contracture.

On physical examination, some degree of mental retardation was evident and she had appearance of wrinkles and aging (Fig.1). Infant had multiple skin folds which were hanging loosely from the cheek and chin like folds of a loose dress. The infant attracted attention due to her normocephalic head, tight abdomen, and small anterior fontanelle, and suture site was palpable (Fig.2).She had a short and vane shaped neck, with ears which were wrinkled found below the normal location. Small eyes (microphthalmia), ptosis, hypertelorism, antimongoloid eyelid groove, long philtrum, an upward hook-shaped nose, and high-arched palate were also observed. Form of chest was pectus carinatum but the sites of the nipples were normal. Spinal column had no scoliosis. There was no organomegaly in abdomen. Ambiguous genital system, similar to the female sex was observed (Figs.3 and 4 On examination of organs, movement limitation of the hip, knee, wrist, and elbow because of bilateral contracture (Fig.5).In the chest radiograph, cardiac enlargement, scattered and diffuse opacities particularly in right lung were observed. In echocardiography, mild to moderate enlargement of both ventricles and severe aortic dilatation with ejection fraction of 60% was found. In brain CT scan, a space-occupying lesion or bleeding in the skull and brain parenchyma was not seen. In abdomen sonography, liver size was normal but had heterogenic state, a hypoechoic band in anterior part of liver was observed and a 11-mm stone in the gallbladder was found. Also in hip joint sonography, congenital dislocation of the hip joint was observed and other sonography findings were normal. In laboratory tests:

BUN: 26, Cr: 0.6, K: 5.2, Na: 134, P: 4, Ca: 9.6, CRP: ++, ESR: 25, U/A: Normal, CBC: WBC: 10400, HCT: 24.2, PMN: 42%, lym: 51%, Mono: 2%, AST: 138, ALT: 55, ALK-P: 861, Bil-T: 0.4, Bil-D: 0.2, PT: 18 Sec

The patient under this condition was referred to a genetic center for consultation. A 46xx compatibility was reported after doing a karyotype test. Considering total clinical and paraclinical finding, SCARF syndrome was diagnosed and the patient was discharged with relative improvement. Then after 10 days, she was hospitalized due to aspiration pneumonia symptoms and sepsis and she died after 4 days.

## Discussion

SCARF syndrome is a very rare syndrome that so far only two cases have been reported in articles. In a report, two cases (both of them male) were found with loose skin, hyper extensive joints, inguinal umbilical hernia, craniosynostosis, pectus carinatum, abnormal spine shape, abnormal face form, thick blade neck, uncertain external genital system, multinodular liver, and mild mental retardation. In our patient, there were a majority of the above symptoms, especially five major symptoms [1,2]. The syndrome is categorised as autosomal dominant or autosomal recessive inheritance. Considering relationship of parents and clinical symptoms of patient the syndrome is an autosomal recessive type. In this case, in addition to the above symptoms, articular disorders especially in the hip, knee, wrist, and elbow because of contracture were observed as well. These problems are usually observed with this syndrome. Since these patients are suffering from mental retardation, in our case, there was no possibility to check this problem completely. It should be mentioned that due to limitation of related articles, there was no possibility of further survey and comparison.
